# Prevalence of Operated Facial Injury in the Department of Oral and Maxillofacial Surgery of a Tertiary Hospital

**DOI:** 10.31729/jnma.4567

**Published:** 2020-01-31

**Authors:** Safal Dhungel, Ashutosh Kumar Singh

**Affiliations:** 1 Department of Oral and Maxillofacial Surgery, College of Medical Sciences and Teaching Hospital, Bharatpur, Chitwan, Nepal

**Keywords:** *facial injuries*, *maxillofacial injuries*, *prevalence*, *trauma*

## Abstract

**Introduction:**

Maxillofacial injury is one of the commonest causes of surgery performed by an oral and maxillofacial surgeon. Socioeconomic conditions, cultural variation, age, and gender affect the etiology of the injury. The study is aimed to find the prevalence of facial injury that is operated by the oral and maxillofacial surgeons in the College of Medical Sciences and Teaching Hospital, Bharatpur, Chitwan, a tertiary hospital.

**Methods:**

A descriptive cross-sectional study was performed using the chart from the hospital registry for the patient being operated under general anesthesia from April 1, 2017, to March 2019. Simple random sampling was done using computer-generated random numbers. Ethical approval was received from the Institutional Review Committee of the hospital. The Data for the reason for surgery, age, age groups etiology, and tissue involvement were analyzed using Statistical Package for the Social Sciences version 20.

**Results:**

Facial injury occupies 378 (71.59%) of the total operation performed by Oral and Maxillofacial surgeon in a tertiary hospital. Soft tissue 196 (52.85%) and facial bone fracture 182 (48.15%) is distributed among the facial injuries. Young adults are commonly affected, and the road traffic accident is the major cause of facial trauma.

**Conclusions:**

Facial injury-related surgeries are more prevalent in the tertiary hospital of Bharatpur.

## INTRODUCTION

Oral and Maxillofacial Surgery (OMFS), the young department of trauma management, is a bridge between dentistry and other medical fields.^1^ Injuries of teeth, facial skeleton, and orofacial soft tissues referred to as maxillofacial injuries are dealt with by oral and maxillofacial surgeons.^2^ Maxillofacial injuries have a close association to traumatic brain injuries hence is seriously attended. Direct procedural expenses and indirect loss because of abstinence from daily activity and reduction in production hampers the society by maxillofacial injuries.^3^

Knowledge about the epidemiology of facial injuries helps to understand the occurrence, cause, and pattern of the injuries thus helping the practitioners for diagnosis and plan for the reconstruction and contributes society for making proper policies.^2–6^

This study is to aim to determine the prevalence of the oro-facial injuries, their distribution, and pattern of injuries among the cases that are attended by the OMFS Department in the College of Medical Sciences Bharatpur, Chitwan.

## METHODS

A descriptive cross-sectional study was performed using the chart from the hospital registry for the patient being operated under general anesthesia by the department of oral and maxillofacial surgery in the College of Medical Sciences, Bharatpur, during April 1, 2017, to March 2019. Ethical approval was received from the Institutional Review Committee of COMS (IRC No. 2019-024). Facial injuries that were managed under local anesthesia were excluded from the study. The sample size was calculated by,

$$\begin{array}{ll} \text{Sample size }(\text{n)} & \text{= }{\text{z}}^{\text{2}}\times \text{p }(1-\text{p)}/{\text{e}}^{\text{2}}\\ & \text{= 1}{\text{.96}}^{\text{2}}\times \text{0.5(1}-\text{0.5)}/\text{0}{\text{.05}}^{\text{2}}\\ & \text{= }384 \end{array}$$

where,

Z = 1.96 at 95% Confidence Interval
p = 0.5 at 50% prevalence assumption of facial injuries among total operated cases
q = 0.5 (1-p)
e = 0.05 at 5% margin of error
Total cases operated by OMFS during the study period (N) = 837
For finite population correction,

$$\begin{array}{ll} \text{Adjusted Sample size } & \text{= n}/\{1\;+\;(\text{n}/\text{N)}\}\\ & \text{= 384}/\{1\;+(384/837)\}\\ & \text{= }264 \end{array}$$

Correcting the sample size, 264 × 2 = 528.

From the hospital registry, patient id number of the cases operated by Oral and Maxillofacial surgery from April 2017 to March 2019 was obtained. From this study population, using simple random sampling (computer-generated random number), the required number of samples were selected for further analysis using SPSS 20. The reason for surgery, age, age groups etiology, and tissue involvement were analyzed.

## RESULTS

In 528 operated sample, 378 (71.59%) cases were of facial injuries. A total of 425 (80.49%) males and 103 (19.51%) females were included in the study. The distribution of reason for surgery is given below (Table 1).

**Table 1:** Distribution of reason for surgery.

Reasons for surgery	n (%)
Facial injuries	378 (71.59)
Pathology	53 (10.04)
Secondary surgery	97 (18.37)
Total	528 (100)

Out of 378 cases of facial injuries, 182 (48.15%) had facial bone fractures, while 196 (51.85%) had complex soft tissue injuries requiring repair under GA/IVA (Table 2).

**Table 2:** Distribution of types of facial injuries.

Facial injuries	n (%)
Bony fracture	182 (48.15)
Soft tissue	196 (51.85)
Total	378 (100)

When the etiology of the trauma was checked, we found the highest cause of trauma to be RTA 226 (59.79%). Fall was the second cause of the trauma of the face (Table 3).

**Table 3:** Distribution of etiology of facial injuries.

Etiology of trauma	n (%)
RTA	226 (59.79)
Fall	63 (16.67)
Assault	49 (12.96)
Sports	15 (3.97)
Animal	14 (3.70)
Other	11 (2.91)
Total	378 (100)

Facial injuries were more prevalent in the third decade (21-30 years), with 141 (37.30%) cases (Table 4).

**Table 4:** Distribution of facial injuries cases in the age range.

Age group	Bony Fracture	Soft Tissue Injuries	Total
<20	42	36	78 (20.63)
21-30	73	68	141 (37.30)
31-40	39	50	89 (23.54)
41-50	17	20	37 (9.79)
>50	11	22	33 (8.73)
Total	182	196	378 (100)

## DISCUSSION

Maxillofacial trauma, commonly encountered in the emergency department, is managed by an oral and maxillofacial surgeon. Trauma is linked with the socioeconomic condition, cultural variation, age and gender along with the inhabitant population in the study area.^7–9^ The pattern of facial injuries varies from soft tissue injury (abrasion, hematoma, laceration and tissue loss) to bony fractures. In our study, male patients had more facial injuries than females. The gender-wise ratio in our study is 5.2:1, which is slightly more than 3:1 in another study.^4,5^ This variation among gender may be because men are involved in outdoor works for earning and living. Also, males are more indulge in alcohol consumption, drinking and driving, and interpersonal violence than females. Whereas in society with male dominance, the ratio rises to 13.1:1.^10^ The average age of the patient with maxillofacial trauma in our study is 30.96 years. The age-wise distribution shows that the third decade has the highest number of facial injuries followed by the fourth, showing accordance with other studies.^4,11,12^ The bony fractures have lower mean age (29.54 years) than the soft tissue injury (32.20 years) which is also similar to study by Prasad et al.^13^ This young predominance of facial injuries maybe because of young male are involved in activities like sports, interpersonal violence, high speed traveling and drinking and driving.^14^ As development occurs etiology for facial injuries also changes.^15^ In our study, the highest cause of facial injuries is RTA (59.79 %), which is followed by a fall (16.67%) physical assault (12.96%). The cause of facial injuries is in developing countries is led by RTA^13^ which is contrary to the developed world.^16,17^ The developing country has poor road facilities and people are always in haste to travel for earning. Crowded public transport, lenient traffic rules, ill-functioning traffic lights may be the reason for more RTA induced facial injuries. Whereas in the developed world road facilities, strict traffic rules, proper surveillance of over speed, and vehicles with proper safety measures are imposed.^17^ Chitwan, developing city, has two major national highway running through the city, also adds reason for more RTA induced facial injuries.

Fall from a height is the second reason for the facial injuries in our study; this may be because of two reasons. Firstly, Chitwan has high hills in its nearby area, and all the cases are transported to Chitwan, and secondly, the forest is dense in the Chitwan district as people in nearby villages are farmers and dependent on firewood for cooking and leaves of trees to feed their animals. During fetching of their necessities, an incident of fall does occur and hence facial of injuries. Physical assaults is a leading cause of facial injuries in developed world,^14,17,18^ while it is third in developing country,^3,19^ and so is in our study. Wild animals attack in the forest while fetching necessities or animals entering the village are major reasons for animal induced injuries, which makes around 3.7% of facial injuries. In our study, unilateral fractures were more common than bilateral (2.8:1). This unilateral to bilateral ratio is very less than the study by the park (14.4:1).^20^ This variation in unilateral vs. bilateral between the two studies may be due to the difference in the etiology of facial fractures. Also, there is no difference in the left side or right side in our study which again contrast the same study.^20^

In this study, we observed the highest percentage of fracture occurred in the mid-face, followed by a lower third of the face. The higher prevalence of the midface fracture is also reported in studies^5,11^ in contrast to other studies where more incidence of fracture in lower facial third is reported.^2,4,13,16^ This higher prevalence of the midfacial injury may be attributed to the wider face and prominent malar bone of Asian people.^21^ Having two major national highway across the city, with buses and coaches running, the impact of the sitting passenger to the seat in front may also be the reason for more midfacial fractures in our study.

Among the fractures, the highest prevalence in our study is of ZMC (27.08%) followed by mandibular fractures (23.75%). In other studies, the prevalence of mandibular fracture ranges from 11.1% to 53.8%, whereas for ZMC, its 2.9% to 33.5%.^2,3,5,9,22^ As the cases are done in GA/IVA only were included in our study, few cases of the mandibular fracture, dentoalveolar, which are done in LA, are excluded, maybe the reason for the higher ZMC fractures than mandibular fracture.

Isolated zygomatic arch, makes less than 5% of total facial fracture,^23^ seen in 3.75% in our study which is between 1.9% and 4.9% in other studies.^4,20^ All isolated nasal bone fractures and upper facial involving the inner table of frontal bone are operated by ENT and neurosurgery department respectively in our institution hence are not taken to consideration.

Among maxillary fractures, Lefort I have the highest prevalence, followed by Lefort II and Lefort III. This decreasing order of the Lefort fracture is also seen in other studies.^4,16,22^ However, the study by Mohajerani^24^ showed a prevalence of Lefort II more than Lefort I.

## CONCLUSIONS

This study concludes that maxillofacial injuries is one of the commonest causes of surgery to be performed by Oral and Maxillofacial Surgeon. A large numbers of population acquiring facial injuries are young adolescents, and RTA is the primary cause. Mid Facial injuries are more prevalent.

## Conflict of Interest

**None.**

## References

[1] Bell RB (2007). The role of oral and maxillofacial surgery in the trauma care center. J Oral Maxillofac Surg.

[2] Majambo MH, Sasi RM, Mumena CH, Museminari G, Nzamukosha J, Nzeyimana A (2013). Prevalence of oral and maxillofacial injuries among patients managed at a teaching hospital in Rwanda. Rwanda J Health Sci.

[3] Abosadegh MM, Saddki N, Al-Tayar B, Rahman SA (2019). Epidemiology of Maxillofacial Fractures at a Teaching Hospital in Malaysia: A Retrospective Study. Biomed Res Int.

[4] Padmanaban SA, Suresh D, Saravanan R, Kavitha PS (2017). Incidence and Prevalence of Maxillofacial Injuries in Government Theni Medical College - Two Years Retrospective Study. Int J Sci Study.

[5] Emodi O, Wolff A, Srouji H, Bahouth H, Noy D, Abu El Naaj I (2018). Trend and demographic characteristics of maxillofacial fractures in level I trauma center. J Craniofac Surg.

[6] Carvalho BLAA, da Silva RS, Galvao EL, Fernandes IA, Falci SGM, dos Santos CRR (2017). Prevalence of Maxillofacial Traumas in a Hospital of the Interior of Brazil. J Dent Health Oral Disord Ther.

[7] Goodfellow M, Burns A (2019). Relation between facial fractures and socioeconomic deprivation in the north east of England. Br J Oral Maxillofac Surg.

[8] Corfield AR, MacKay DF, Pell JP (2016). Association between trauma and socioeconomic deprivation: a registry-based, Scotland-wide retrospective cohort study of 9,238 patients. Scand J Trauma Resusc Emerg Med.

[9] Batista AM, Marques LS, Batista AE, Falci SG, Ramos-Jorge ML (2012). Urban-rural differences in oral and maxillofacial trauma. Braz Pral Res.

[10] Al-Hassani A, Ahmad K, El-Menyar A, Abutaka A, Mekkodathil A, Peralta R (2019). Prevalence and patterns of maxillofacial trauma: a retrospective descriptive study. Eur J Trauma Emerg Surg.

[11] Pungrasmi P, Haetanurak S (2017). Incidence and etiology of maxillofacial trauma: a retrospective analysis of King Chulalongkorn Memorial Hospital in the past decade. Asian Biomed.

[12] Kapoor P, Kalra N (2012). A retrospective analysis of maxillofacial injuries in patients reporting to a tertiary care hospital in East Delhi. Int J Crit Illn Inj Sci.

[13] Prasad C, Narayanan MBA, Parimala V, Vijjaykanth M (2018). Prevalence and pattern of maxillofacial trauma in North Chennai: A retrospective study. J Indian Assoc Public Health Dent.

[14] Romanet I, Graillon N, Le Roux MK, Guyot L, Chossegros C, De Boutray M (2019). Hooliganism and maxillofacial trauma: The surgeon should be warned. J Stomatol Pral Maxillofac Surg.

[15] Martinez AY, Como JJ, Vacca M, Nowak MJ, Thomas CL, Claridge JA (2014). Trends in maxillofacial trauma: a comparison of two cohorts of patients at a single institution 20 years apart. J Pral Maxillofac Surg.

[16] Pillay L, Mabongo M, Buch B (2018). Prevalence and aetiological factors of maxillofacial trauma in a rural district hospital in the Eastern Cape. South African Dental Journal.

[17] Boffano P, Roccia F, Zavattero E, Dediol E, Uglesic V, Kovacic Z (2015). European Maxillofacial Trauma (EURMAT) project: a multicentre and prospective study. J Craniomaxillofac Surg.

[18] Alvi A, Doherty T, Lewen G (2003). Facial fractures and concomitant injuries in trauma patients. Laryngoscope.

[19] Tripathi GM, Sharma D, Gaharwar APS, Gupta R, Shukla D, Shukla V (2016). Analysis of Prevalence and Pattern of Zygomatic Complex Fractures in North-Eastern Part of Madhya Pradesh, India. Int J Contemp Med Res.

[20] Park KP, Lim SU, Kim JH, Chun WB, Shin DW, Kim JY (2015). Fracture patterns in the maxillofacial region: a four-year retrospective study. J Korean Assoc Oral Maxillofac Surg.

[21] Mahatumarat C, Rojvachiranonda N (2003). Reduction malarplasty without external incision: a simple technique. Aesthetic Plast Surg.

[22] Gadre KS, Halli R, Joshi S, Ramanojam S, Gadre PK, Kunchur R (2013). Incidence and Pattern of Cranio-Maxillofacial Injuries: A 22 year Retrospective Analysis of Cases Operated at Major Trauma Hospitals/Centres in Pune, India. J Maxillofac Oral Surg.

[23] Swanson E, Vercler C, Yaremchuk MJ, Gordon CR (2012). Modified Gillies approach for zygomatic arch fracture reduction in the setting of bicoronal exposure. J Craniofac Surg.

[24] Mohajerani SH, Asghari S (2011). Pattern of midfacial fractures in Tehran, Iran. Dent Traumatol.

